# Abnormal elevation of myocardial necrosis biomarkers after coronary artery bypass grafting without established myocardial infarction assessed by cardiac magnetic resonance

**DOI:** 10.1186/s13019-017-0684-3

**Published:** 2017-12-29

**Authors:** Fernando Teiichi Costa Oikawa, Whady Hueb, Cesar Higa Nomura, Alexandre Ciappina Hueb, Alexandre Volney Villa, Leandro Menezes Alves da Costa, Rodrigo Morel Vieira de Melo, Paulo Cury Rezende, Carlos Alexandre Wainrober Segre, Cibele Larrosa Garzillo, Eduardo Gomes Lima, Jose Antonio Franchini Ramires, Roberto Kalil Filho

**Affiliations:** Instituto do Coracao (InCor), Hospital das Clinicas HCFMUSP, Faculdade de Medicina, Universidade de São Paulo, São Paulo, SP Brazil

**Keywords:** Myocardial infarction, Biomarkers, Coronary bypass surgery, Periprocedural, Troponin

## Abstract

**Background:**

The diagnosis of peri-procedural myocardial infarction is complex, especially after the emergence of high-sensitivity markers of myocardial necrosis.

**Methods:**

In this study, patients with normal baseline cardiac biomarkers and formal indication for elective on-pump coronary bypass surgery were evaluated. Electrocardiograms, cardiac biomarkers, and cardiac magnetic resonance imaging with late gadolinium enhancement were performed before and after procedures. Myocardial infarction was defined as more than ten times the upper reference limit of the 99th percentile for troponin I and for creatine kinase isoform (CK-MB) and by the findings of new late gadolinium enhancement on cardiac magnetic resonance. We assessed the release of cardiac biomarkers in patients with no evidence of myocardial infarction on cardiac magnetic resonance.

**Results:**

Of 75 patients referred for on-pump coronary bypass surgery, 54 (100%) did not have evidence of myocardial infarction on cardiac magnetic resonance. However, all had a peak troponin I above the 99th percentile; 52 (96%) had an elevation 10 times higher than the 99th percentile. Regarding CK-MB, 54 (100%) patients had a peak CK-MB above the 99th percentile limit, and only 13 (24%) had an elevation greater than 10 times the 99th percentile. The median value of troponin I peak was 3.15 (1.2 to 3.9) ng/mL, which represented 78.7 times the 99th percentile.

**Conclusion:**

In this study, different from CK-MB findings, troponin was significantly increased in the absence of myocardial infarction on cardiac magnetic resonance. Thus, CK-MB was more accurate than troponin I for excluding procedure-related myocardial infarction. These data suggest a higher troponin cutoff for the diagnosis of coronary bypass surgery related myocardial infarction.

**Clinical trial registration:**

http://www.isrctn.com/ISRCTN09454308. Registered 08 May 2012.

## Background

Myocardial necrosis biomarkers are frequently elevated after cardiac revascularization procedures. However, the diagnosis of acute myocardial infarction (MI) after a revascularization procedure is still a controversial issue. This inability to diagnose MI makes it more difficult to establish a specific therapeutic strategy. With the appearance of high-sensitivity troponins, a myriad of false-positive diagnoses for myocardial infarction have emerged. In 2000 and 2007 in an attempt to standardize the criteria for diagnosing MI, the European Society of Cardiology, the American College of Cardiology, the American Heart Association, and the World Heart Federation formed a joint task force to address this issue, but the task force was unable to make a satisfactory decision. Therefore, the problem still remained. To reduce diagnostic mistakes, in 2012, this same group arbitrarily raised the cutoff point to 10 times the 99th percentile, but with no solid scientific basis for doing so [1]. Troponin (cTnI) and the creatine kinase isoform (CK-MB) do not reflect, alone, the occurrence of MI related to occlusion of the graft or native artery or varying degrees of myocardial injury. Release of myocardial necrosis markers may be related to incomplete myocardial protection; reperfusion injury; a systemic inflammatory state, including inevitable postsurgical trauma; the handling of intramyocardial vessels; and cardiac defibrillator use [2, 3]. Cardiac troponin may also be increased when nonsurgical damage is present, such as sepsis and thromboembolic phenomena [1]. cTnIs have also been found elevated in athletes after marathons [4]. This makes the identification of small areas of injury very difficult to assess in clinical practice [5].

Parallel to the increased sensitivity of troponin assays, imaging methods have achieved better accuracy for exclusion of the diagnosis of myocardial infarction. Thus, due to the limitations on the interpretation of biomarkers after coronary artery bypass grafting (CABG) and the difficulty of excluding MI, cardiac magnetic resonance imaging (CMR) has enabled a more detailed evaluation of the myocardium.

Therefore, in this study, we aimed to examine the release of biomarkers after CABG in patients with no evidence of late enhancement on CMR.

## Patients and methods

Details of the study design, protocol, patient selection, and inclusion criteria have been previously reported [6]. Briefly, patients with preserved left ventricular function and angiographic coronary artery stenosis of more than 70% confirmed by a visually reviewed document, and with multiple-vessel involvement, and documented ischemia were included. Stress testing or evaluation of stable angina according to the Canadian Cardiovascular Society guidelines (Class II or III) established the presence of ischemia. All patients were candidates for on-pump coronary artery bypass grafting (ONCAB). Patients were excluded if they had undergone any previous mechanical interventions, and had experienced recent thromboembolic events, systemic inflammatory disease, or kidney failure.

### Trial outcomes

The primary outcome was the occurrence of MI based on the release of the biomarkers, cTnI and CK-MB, in patients with no late gadolinium enhancement (LGE) assessed by CMR.

## Methods

### Surgical technique

In accordance with current best practices, the same team of surgeons with experience in ONCAB performed the procedures. Surgical access to the heart was through a standard median sternotomy in all cases. All incisions and closure techniques were performed in the same way in all patients to limit variability among patients.

### CMR protocol

CMR was performed before and after the surgical procedure. CMR, considered the gold standard, allows high-precision assessment that is reproducible in the same test. Recent studies indicate that CMR detects MI very accurately and provides results similar or superior to results with radionuclide imaging [7–10].

All patients underwent CMR 2 days before the intervention and 6 days after each invasive procedure during the hospitalization period. A 1.5-T Achieva Magnetic Resonance scanner (Philips Healthcare, Andover, MA) was used. Steady-state free precession cine images were acquired in 2 long-axis (2 and 4 chambers) views and 8 to 10 short-axis views of the left ventricle. Contrast-enhanced images were acquired in long- and short-axis planes identical to the cine images. Typical voxel size was 1.6 × 2.1 × 8 mm, with a reconstruction matrix of 528 and a reconstructed voxel size of 0.6 mm. The method for acquiring and analyzing CMR was standardized in our service and was reproduced according to conventional techniques [11, 12]. Delayed enhancement on CMR was performed with a phase-sensitive inversion recovery (PSIR) sequence (repetition time 6.1 ms echo time 3.0 ms, voxel size 1.6 × 2.1x8mm, flip angle 25^o^) following a 5-min time delay after the administration of 0.1 mmol/kg contrast agent (GadoteratemeglumineGd-DOTA™, Guerbet SA, France). Images were acquired in 2 long-axis planes and in a short-axis stack covering the entire left ventricle. The inversion time was meticulously adjusted throughout the acquisition to obtain optimal nulling of remote normal myocardium. The slice thickness at the apex was reduced to 5 mm to avoid a partial volume effect. MI was defined as the identification of hyper-enhancement in the myocardium on CMR. Infarcted regions exhibit this phenomenon, which might be due to an increased volume of distribution of the contrast agent, because of rupture of myocyte membranes and slow contrast washout [10].

#### CMR analysis

All areas of late gadolinium-diethylene-triamine-pentaacetic acid (DTPA) hyper-enhancement were quantified by 2 experienced observers who interpreted the LGE while blinded to the interventional technique and biochemical data. When measurements differed, a third observer performed a review, and a consensus was obtained. Hyper-enhanced pixels were defined as those with image intensities exceeding 2 standard deviations greater than the mean of image intensities in a remote myocardial region in the same image. Pre-intervention and post-intervention scans were read side by side in both surgical techniques, with and without extracorporeal circulation.

### Biochemistry

All blood samples for measurement of cTnI and CK-MB were collected immediately before and 6, 12, 24, 36, 48, and 72 h after on-pump CABG. The surgeon and clinical team were blinded to the CK-MB or cTnI data. All samples were centrifuged at 3000 rpm for 20 min and analyzed within 2 h after collection. Analyses of cTnI and CK-MB were performed using an ADVIA Centaur immunoassay analyzer (Siemens Health Care Diagnostics, Tarrytown, NY). According to the manufacturer, the lower limit of detection of cTnI using the high-sensitivity Ultra kit is 0.006 ng/mL, and the 99th percentile upper reference limit (URL) is 0.04 ng/mL. The assay precision represented by the percentage coefficient of variation is 10% at 0.03 ng/mL. The detection limit of the CK-MB mass kit (Acute Care™ CK-MB assay Siemens™) is 0.18 ng/mL. Cutoff values at the 99th percentile are 3.8 ng/mL for women and 4.4 ng/mL for men. The coefficients of variations for CK-MB mass, as specified by the manufacturer, are 3.91% at 3.55 ng/mL and 3.67% at 80.16 ng/mL.

### Definition of CABG-related MI

According to the Third Universal Definition [1], MI type V is defined as an elevation of more than 10 times the 99th percentile during the first 48 h after CABG. Patients with normal baseline cTnI concentrations plus any of the following criteria were considered to have experienced an MI: (1) new pathologic Q waves or new left bundle-branch block (LB-BB), (2) angiographically documented new graft or new native coronary occlusion, or (3) imaging evidence of new loss of viable myocardium or new regional wall motion abnormality.

### Electrocardiograms

Twelve-lead electrocardiograms (EKG) were obtained from each patient immediately before and 6, 12, 24, and 36 h after CABG. For the identification of new Q waves, we used the Minnesota code, which is used extensively in epidemiology studies and large-scale clinical trials [13].

### Ethics committee approval

All patients provided written informed consent and were assigned to a treatment group. The Ethics Committee of the Heart Institute of the University of São Paulo Medical School, São Paulo, SP, Brazil, approved the trial. All procedures were performed in accordance with the Declaration of Helsinki.

### Statistical analysis

Values are expressed as mean and standard deviation or median and interquartile range, as appropriate. The paired-sample *t* test and the unpaired-sample *t* test were used to compare means within the study group or between subgroups. The chi-square and the Fisher exact tests were used for comparison of discrete variables. Continuous variables without normal distribution were compared using the Mann-Whitney U test, and correlation between such variables was made with the Spearman rank test. Values of *p* < 0.05 were considered statistically significant.

## Results

**Fig. 1:**
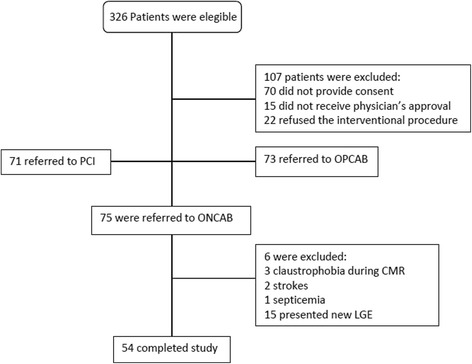
Consolidated Standards of Reporting Trials (CONSORT) diagram. (CMR = cardiac magnetic resonance; LGE = late gadolinium enhancement; ONCAB = on-pump coronary artery bypass; OPCAB = off-pump coronary artery bypass; PCI = percutaneous coronary intervention)

Between March 2012 and April 2014, 326 consecutive patients who met the inclusion criteria were screened. Of these patients, 107 (32.8%) were excluded (Fig. 1). Of the 219 remaining patients, 148 were referred for CABG (75 ONCAB and 73 OPCAB [off-pump coronary artery bypass]), and 71 patients were referred for PCI (percutaneous coronary intervention). Of the 75 ONCAB patients enrolled in this study, 21 were excluded and 54 completed the study protocol. These 54 patients had no evidence of MI on CMR assessed by LGE. The main reasons for exclusion of the patients are presented in Fig. 1.

The clinical, demographic, and angiographic characteristics are summarized in Table 1. The mean age was 61.3 (± 8.3) years, and 39 (72.2%) were male. In addition, 24 (44.4%) patients had a diagnosis of type 2 diabetes mellitus and 13 (24.1%) had a history of myocardial infarction. Regarding smoking, 18 patients (33.34%) stopped smoking during the inclusion period of the study. The angiographic screening showed that 17 (31.5%) patients had stenosis of the left main coronary artery, 43 (80%) had obstructive lesions in 3 epicardial branches, and 11 (20.4%) had a concomitant bi-arterial obstructive pattern. Additionally, the mean SYNTAX Score was 28. Anginal symptoms were present in 47 (87%) patients, and 15 (27.8%) had grade III angina, according to the Canadian Cardiovascular Society (CCS) scale. Left ventricular ejection fraction was assessed by CRM performed before the procedure and averaged 66 ± 8.6 (Table1).

**Table 1:** Clinical, demographic and angiographic characteristics of study population

	*N* = 54
Age, y	61.3 ± 8.3
Male, *n* %	39 (72.2)
Diabetes mellitus, *n* (%)	24 (44.4)
Hypertension, *n* (%)	48 (88.9)
Current smoker, *n* (%)	6 (11.1)
Former smoker, *n* (%)	18 (33.3)
Previous myocardial infarction, *n* (%)	13 (24)
Angina, *n* (%)	47 (87)
Angina CCS III-IV, *n* (%)	15 (27.8)
Total cholesterol, mg/dL	161 ± 42.4
LDL cholesterol, mg/dL	93 ± 37
HDL cholesterol, mg/dL	39 ± 13
Triglycerides, mg/dL	160 ± 148
Left main disease, *n* (%)	17 (31.5)
Double-vessel disease, *n* (%)	11 (20.4)
Triple-vessel disease, *n* (%)	43 (80)
SYNTAX Score,	28 ± 10
Ejection fraction, median %	66 ± 8.6

*N* Number of patients, *CCS* Canadian Cardiovascular Society, *LDL* low-density lipoprotein, *HDL* high-density lipoprotein

### Cardiac biomarkers

**Fig. 2:**
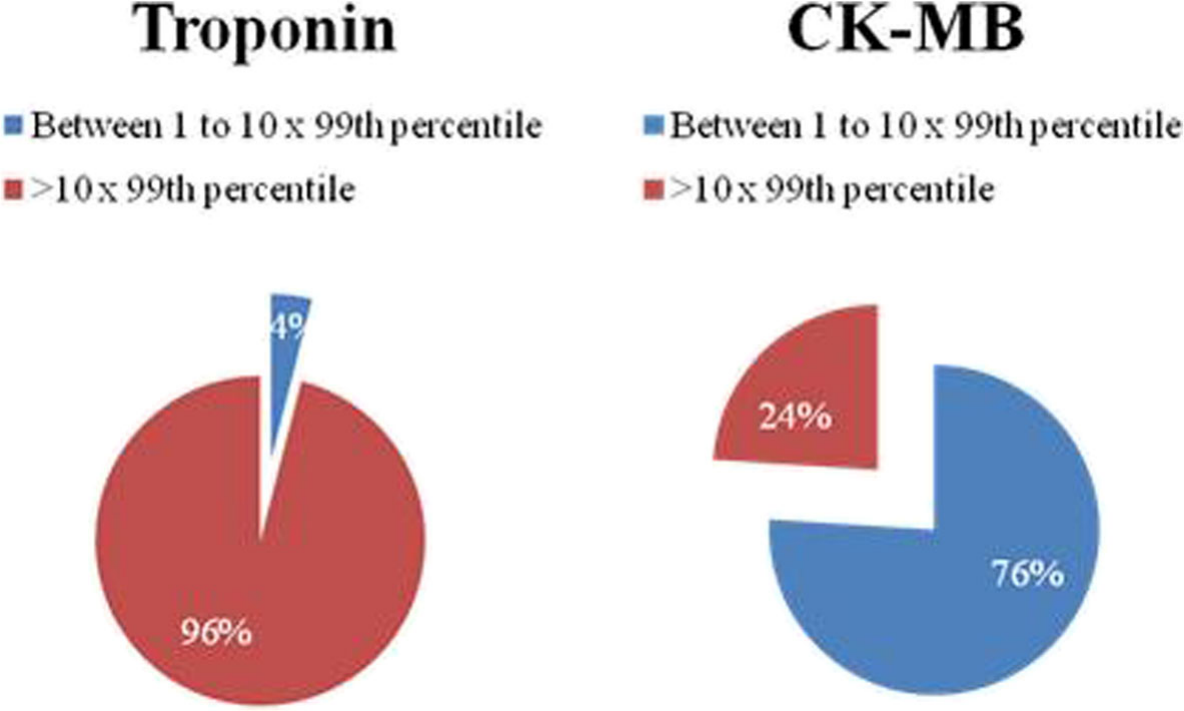
Percentage of patients with elevated biomarkers according to cutoff levels with no evidence of LGE on CMR

The median value of troponin peak was 3.15 (2.0 to 4.9) ng/mL, which corresponds to 78.7 times the 99th percentile. Two (4%) patients had elevation just above the 99th percentile, and 52 (96%) remaining patients had elevation above 10 times the 99th percentile. There were no patients with a cTnI value below the 99th percentile after the surgical procedure (Fig. 2A).

Regarding CK-MB peak, the median value was 23.0 ng/mL (14.2 to 38.3 ng/mL). Additionally, 41 (76%) patients had elevation above the 99th percentile, and 13 (24%) had an increase higher than 10 times the 99th percentile (Fig. 2B).

**Fig. 3:**
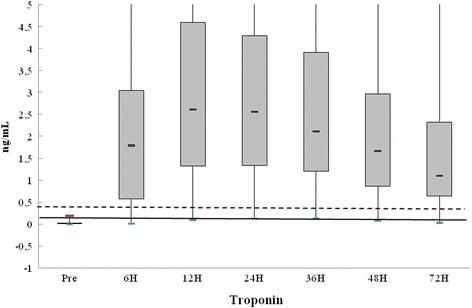
Distribution of cTnI before and after surgical procedure. Continuous line shows the 99th percentile and dashed line 10 times the 99th percentile

The pattern of cTnI elevation in each moment of evaluation after surgery is shown in the chart below (Fig. 3). Values for cTnI above 10 times the 99th percentile are constant over the measurement time in almost the entire sample.

The comparisons of the levels of cTnI in the different periods after the procedure showed a statistically significant difference, *p* < 0.001 in all groups.

**Fig. 4:**
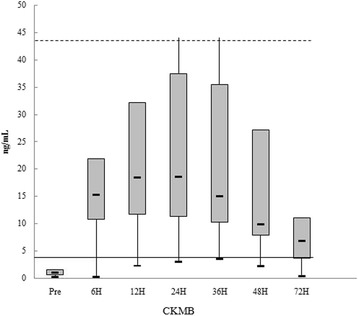
Distribution of CK-MB before and after surgical procedure. Continuous line shows the 99th percentile and dashed line 10 times the 99th percentile

The pattern of CK-MB elevation in each moment after surgery is shown in the chart below, respectively (Fig. 4). Only a small part of the sample reached values above the 99th percentile.

The comparisons of CK-MB values in the different periods after the procedure showed statistically significant differences, *p* < 0.001 in both groups.

### Electrocardiogram

All of the 54 patients who were free of late enhancement on the CMR after the procedure and who were selected for this study underwent ECG at entry and sequentially. None of them had a new bundle-branch block, ischemic ST-segment, new pathologic Q wave conduction disorders, or a new Q wave after the procedure.

### Renal function

All patients had preserved glomerular filtration rate on admission. Sequential measures of renal function indicated that no loss of this function occurred.

## Discussion

In this prospective trial based on current guidelines, we found distinct results when cTnI and CK-MB were simultaneously analyzed in patients after surgical myocardial procedures. All patients had elevated cTnI above the 99th percentile after surgery, with the majority having more than 10 times, reaching an average of 70 times this threshold. Conversely, we found the release of CK-MB predominantly below the recommended threshold of 10 times the 99th percentile. Therefore, our findings conflict with the recommendations of the 2012 European Society of Cardiology/American College of Cardiology/American Heart Association/World Heart Foundation Joint Task Force for the diagnosis of myocardial infarction after surgical revascularization.

In this scenario, the EKG remained similar before and after interventions independently of the release of biomarkers. In addition, CMR likewise remained unchanged, without a new delayed enhancement after the procedure.

Over the last decade, elective CABG has progressed to a very standardized and safe surgical procedure with low mortality and low rates of myocardial events [14]. Thus, the present study focused on the possible reasons why our patients, after ONCAB, had elevated troponin above MI levels without the appearance of late enhancement in cardiac magnetic resonance imaging. As we know, perioperative elevation of specific cardiac biomarkers may be due to MI, but may also be associated with routine cardiac surgical procedures.

A study aimed at identifying the release of biomarkers following myocardial revascularization conducted by Pegg et al. [15] identified excessive troponin release in the absence of late enhancement by CMR. On the other hand, they noted that CK-MB behaved as foreseen by the current guideline. Thus, their results confirm the findings of the present study. Likewise, Van Gaal et al. [16], in a study comparing cTnI elevation and appearance of new late enhancement in CMR after CABG, found late enhancement in 28.1% of their patients. However, troponin elevation was found in 100% of patients based on the definition of myocardial infarction by the third Task Force [1]. Similar results were observed by Fellahi et al. [17] who, in an accurate analysis, found 14% troponin elevation in the absence of late enhancement. This lower percentage of discordance was probably due to the use of less-sensitive troponin assays. Wang et al. [18] applying EKG and echocardiography as the gold standard to detect AMI after CABG identified 21% of patients with new regional changes in wall motion on echocardiography without the corresponding change on EKG. The echocardiographic findings were consistent with the Troponin Task Force definition. Conversely, EKG data from our study were consistent with CK-MB release and discordant with troponin release.

The evident release of cTnI in the absence of myocardial necrosis is still questioned in the literature. As a confounding factor, advances in cTnI accuracy after high-sensitivity cTnI onset have been recently observed [18]. It can be postulated that this increase in sensitivity may be related to the power to detect changes in myocyte membrane permeability, which may result from non-physiological intraoperative events, contributing to the increase of cTnI plasma levels in the cytosol even in the absence of necrotic damage (Type 5 troponin elevation) [11, 19]. Therefore, a possible deleterious effect of extracorporeal circulation may contribute to the occurrence of discrete and “diffuse” myocardial damage. This damage can compromise subcellular structures that are difficult to identify, evidencing a clear limitation of CMR analysis.

With the emergence of high-sensitivity troponins, the relationship between the increase in the sensitivity thereof and the increasing rise in false diagnoses has already been described [2]. Currently, there is extensive discussion among manufacturers about the heterogeneity of their troponin kits and the influence of laboratory practices on the use of these kits. The multiplicity of troponin kits, each having different reference values, which use different reagents, different epitopes to bind antibodies, and different incubation times, leads to great difficulty in finding uniformity in the acquired information and studies [20–22]. Unlike that observed with CK-MB, the lack of standardization for calibration of the different tests for assessment of cTnI precludes the establishment of a universal threshold cutoff for the 99th percentile [20].

### Clinical implications

Assuming that cardiac biomarkers have limited diagnostic accuracy in myocardial necrosis, the challenges faced for the establishment of definitive values for the diagnosis of myocardial damage include new cutoff values for cTnI. Furthermore, the diagnosis of this condition cannot be exclusively based on cardiac biomarkers or EKG. It is reasonable to include CMR in the set of tools for the accurate diagnosis of procedure-related myocardial injury.

## Conclusions

In this study, different from CK-MB findings, troponin was significantly increased in the absence of myocardial infarction on cardiac resonance imaging. Thus, CK-MB was more accurate than cTnI for excluding procedure-related MI. These data suggest a higher troponin cutoff for the diagnosis of CABG-related MI.
